# Emvododstat, a Potent Dihydroorotate Dehydrogenase Inhibitor, Is Effective in Preclinical Models of Acute Myeloid Leukemia

**DOI:** 10.3389/fonc.2022.832816

**Published:** 2022-02-09

**Authors:** Arthur Branstrom, Liangxian Cao, Bansri Furia, Christopher Trotta, Marianne Santaguida, Jason D. Graci, Joseph M. Colacino, Balmiki Ray, Wencheng Li, Josephine Sheedy, Anna Mollin, Shirley Yeh, Ronald Kong, Richard Sheridan, John D. Baird, Kylie O’Keefe, Robert Spiegel, Elizabeth Goodwin, Suzanne Keating, Marla Weetall

**Affiliations:** ^1^ Research, PTC Therapeutics, Inc., South Plainfield, NJ, United States; ^2^ Notable Labs, Foster City, CA, United States; ^3^ InSeption Group, Lansdale, PA, United States; ^4^ Clinical, PTC Therapeutics, Inc., South Plainfield, NJ, United States; ^5^ Commercial, PTC Therapeutics, Inc., South Plainfield, NJ, United States; ^6^ Scientific Writing, PTC Therapeutics, Inc., South Plainfield, NJ, United States

**Keywords:** AML, DHODH, differentiation, emvododstat, PTC299, pyrimidine nucleotide *de novo* synthesis, dihydroorotate dehydrogenase

## Abstract

Blocking the pyrimidine nucleotide *de novo* synthesis pathway by inhibiting dihydroorotate dehydrogenase (DHODH) results in the cell cycle arrest and/or differentiation of rapidly proliferating cells including activated lymphocytes, cancer cells, or virally infected cells. Emvododstat (PTC299) is an orally bioavailable small molecule that inhibits DHODH. We evaluated the potential for emvododstat to inhibit the progression of acute myeloid leukemia (AML) using several *in vitro* and *in vivo* models of the disease. Broad potent activity was demonstrated against multiple AML cell lines, AML blasts cultured ex vivo from patient blood samples, and AML tumor models including patient-derived xenograft models. Emvododstat induced differentiation, cytotoxicity, or both in primary AML patient blasts cultured ex vivo with 8 of 10 samples showing sensitivity. AML cells with diverse driver mutations were sensitive, suggesting the potential of emvododstat for broad therapeutic application. AML cell lines that are not sensitive to emvododstat are likely to be more reliant on the salvage pathway than on *de novo* synthesis of pyrimidine nucleotides. Pharmacokinetic experiments in rhesus monkeys demonstrated that emvododstat levels rose rapidly after oral administration, peaking about 2 hours post-dosing. This was associated with an increase in the levels of dihydroorotate (DHO), the substrate for DHODH, within 2 hours of dosing indicating that DHODH inhibition is rapid. DHO levels declined as drug levels declined, consistent with the reversibility of DHODH inhibition by emvododstat. These preclinical findings provide a rationale for clinical evaluation of emvododstat in an ongoing Phase 1 study of patients with relapsed/refractory acute leukemias.

## Introduction

Acute myeloid leukemia (AML) is typified by clonal proliferation and reduced cellular differentiation, resulting in the accumulation of undifferentiated cells with the capacity to self-renew (1, 2). The disease is heterogenous including many disparate subtypes resulting from a variety of genetic alterations in blood cell precursors through chromosomal abnormalities or specific gene mutation (3). Furthermore, subclones that differ genetically often exist within the same patient (4, 5). AML is the second most common leukemia in adults, with the highest incident rate in patients >65 years of age and accounts for the highest percentage of deaths (approximately 62%) of all leukemias (6). As of 2016, the estimated median overall survival for AML is 8.5 months, and the 2- and 5-year overall survival rates are 32% and 24%, respectively (7).

The heterogeneity of AML presents challenges for developing therapies that may be effective against the disease in terms of response to treatment, drug resistance, and disease relapse (2). Broadly, treatment of AML can target the disease *via* 2 processes: promoting cell death of blast cells and/or inducing terminal differentiation ending the ability of self-renewal (proliferation).

The treatment landscape for AML has changed dramatically over the past few years due to a greater understanding of the molecular pathogenesis of the disease and the development of novel targeted therapies (8). However, despite advances in the understanding of the molecular heterogeneity of AML, overall outcomes remain poor (2). All-Trans Retinoic Acid) plus arsenic trioxide without chemotherapy is used to target the promyelocytic leukemia-retinoic acid receptor α chimeric fusion protein-associated acute promyelocytic leukemia, a subtype of AML. Response rates with this non-toxic therapy are more than 95% and long-term remission rates are more than 80% (9). However, this therapy has not been effective against other subtypes of AML. A treatment that would block proliferation and promote myeloid differentiation in the absence of cytotoxicity would be of great benefit for treating other AML subtypes.

Isocitrate dehydrogenase (IDH)1 and IDH2 inhibitors, ivosidenib and enasidenib, which promote myeloid precursor differentiation, are approved for the treatment of adult relapsed or refractory AML in patients with mutant IDH1 and mutant IDH2, respectively (10). While these agents are efficacious and well tolerated, monotherapy has been associated with resistance. Accordingly, ongoing clinical trials are evaluating these drugs in combination with hypomethylating agents or standard chemotherapy (11). In addition, these molecules are limited as they are effective in only the subset of patients with IDH1/2 mutations (approximately 15% of all AML patients) (12). Other therapeutic efforts to induce myeloid differentiation in AML have been largely unsuccessful.

**Figure 1:**
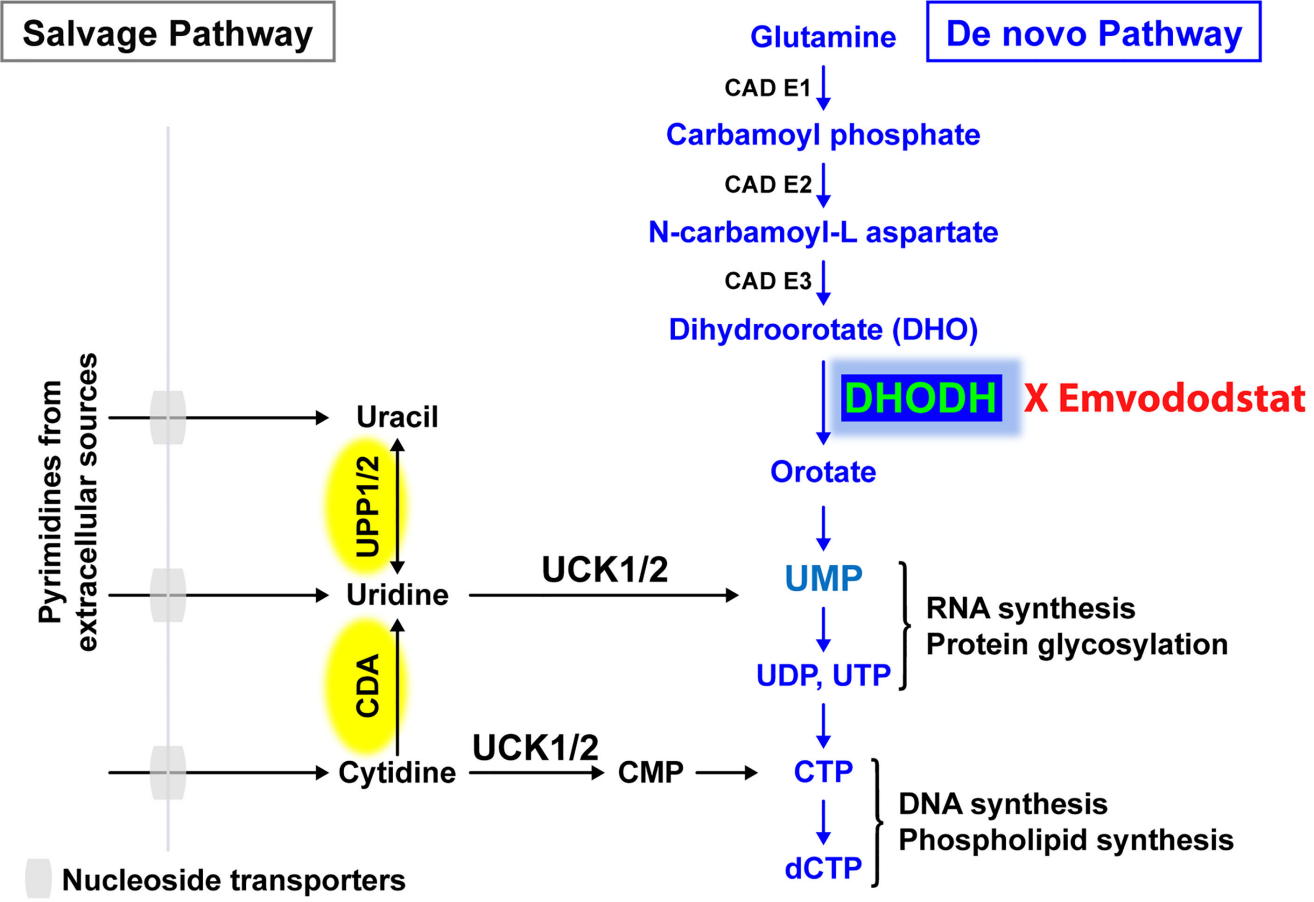
The salvage pathway recycles pre-existing nucleotides from food or other extracellular sources. In rapidly proliferating cells such as AML blast cells, the salvage pathway is not sufficient. Adapted from (13).

Recently, the *de novo* pyrimidine synthesis pathway has been recognized as a potential therapeutic target for AML and other diseases caused by rapidly proliferating cells, with a particular focus on dihydroorotate dehydrogenase (DHODH), the rate-limiting enzyme in this pathway (**Figure 1**) (14, 15). DHODH is expressed in all tissues examined, including the lung, heart, and liver. The enzyme is located on the inner membrane of mitochondria and catalyzes the dehydrogenation of dihydroorotate (DHO) to orotic acid, which is required for the production of pyrimidine nucleotides including cytidine triphosphate (CTP) and uridine triphosphate (UTP). In rapidly dividing cells that rely on *de novo* pyrimidine nucleotide synthesis, depletion of pyrimidine nucleotides through DHODH inhibition results in antiproliferative effects leading to G1/S phase cell cycle arrest and subsequent differentiation or cell death. Proliferating T cells, leukemic or cancer cells, and virus-infected cells all have increased need for pyrimidine nucleotides and thus are more reliant on *de novo* synthesis.

Resting differentiated cells obtain pyrimidine nucleotides *via* the salvage pathway, which utilizes the degradation of RNA and DNA, and are not dependent on *de novo* pyrimidine nucleotide synthesis. It has been hypothesized that malignant cells have a higher requirement for pyrimidine nucleotides and a lower tolerance for pyrimidine nucleotide “starvation”, resulting in a greater dependence on DHODH than do healthy cells (15). Prolonged periods of pyrimidine starvation result in the inhibition of malignant cell proliferation, promotion of differentiation, or death without affecting resting healthy differentiated cells, thus providing a basis for selective activity and a high therapeutic window (16).

Many AML cell lines are sensitive to pyrimidine nucleotide starvation, and DHODH inhibitors have demonstrated preclinical antileukemic activity in a broad range of settings (12, 15, 17, 18). Inhibition of DHODH can result in reduction of myeloid blast cell proliferation, increased blast cell differentiation, and/or AML cell death (12, 17).

Emvododstat (PTC299) is an orally bioavailable small molecule that directly binds to DHODH, inhibiting its enzymatic activity (13). Treatment of cultured cells with emvododstat results in the reduction of DHODH activity, leading to decreased levels of pyrimidine nucleotides and increased levels of DHO, the substrate for the enzyme (13). For cells that are reliant on *de novo* pyrimidine nucleotide biosynthesis, this inhibition results in a reduction in cytokine production (13, 18), cell proliferation, differentiation, and/or cell death. Emvododstat-mediated inhibition of cellular proliferation can be overcome by adding exogenous pyrimidine nucleosides (i.e., cytidine and uridine) but not purine nucleosides (i.e., adenosine and guanosine), consistent with DHODH inhibition (13).

Prior work demonstrated that a diverse array of leukemia cell lines are sensitive to emvododstat. A greater percentage of AML lines (71%) were susceptible to the drug compared with solid tumor cell lines (20%) (13). Emvododstat has greater activity compared with standard-of-care agents for the treatment of AML in studies of murine xenograft models using established leukemia cell lines, including MOLM-13, THP-1, Jurkat-BCL, and HL60 cells. Importantly, in clinical trials with more than 300 healthy volunteers and cancer patients, emvododstat demonstrated a favorable pharmacokinetic profile (19) (PTC Therapeutics, unpublished data).

The purpose of the study described herein was to characterize further the effect of emvododstat on the *de novo* pyrimidine biosynthesis pathway and myeloid blast cell survival and differentiation. These results will provide an understanding of the potential clinical utility of emvododstat for the treatment of AML.

## Methods

### General Methods

All studies involving animals were performed in accordance with the American Association for Accreditation of Laboratory Animal Care (AAALAC) guidelines and with the oversight of the animal use and care committees at Rutgers University.

Test products were obtained from the following sources: emvododstat, PTC-371, and PTC-868, PTC Therapeutics; dimethyl sulfoxide (DMSO), uridine, and brequinar sodium, Sigma-Aldrich Co LLC, MO, USA; 5-azacytidine, Selleck Chemicals, TX, USA; cytarabine, Cayman Chemical, MI, USA; teriflunomide (A771726), Abcam, Cambridge, UK.

### Cell Line Sensitivity Studies

A total of 12 leukemia/lymphoma cell lines were tested for sensitivity to emvododstat and were subsequently evaluated for changes in the levels of specific metabolites. These cell lines (U937, TF-1, Sup-T1, Sup-B15, RS4;11, MV4-11, Molt4, K562, Jurkat, THP-1, and HL60) were obtained from American Type Culture Collection (ATCC; Manassas, Virginia) and cultured using methods provided by ATCC. MOLM 13 cells were a generous gift of Kensuke Kayamori (Saga University).

Briefly, K562 cells were plated in 96-well plates at 5x10^3^ cells/well, HL60 at 3.5x10^4^ cells/well and the other 10 lines were plated at 1x10^4^ cells/well. Cells were treated with increasing concentrations of emvododstat (4.57 nM to 10,000 nM) to determine the concentration resulting in 50% reduction in cell viability as measured by ATP levels (IC_50_; i.e., the concentration that reduces ATP levels by 50%) for each cell line. Cells were assessed for viability after 72 hours using CellTiter-Glo^®^ Luminescent Cell Viability Assay Kit (Promega). The IC_50_ values were calculated using a dose response curve fitted using a nonlinear regression model with a sigmoidal dose response.

### *In Vitro* Metabolite Studies to Measure DHO and N-Carbamoyl L-Aspartate

The same 12 leukemia/lymphoma cell lines were grown in suspension culture in the appropriate growth medium. Prior to treatment, cells were sedimented and resuspended in medium supplemented with fetal bovine serum. T175 Flasks (4x10^7^ cells/flask) were treated with 1 µM emvododstat or 0.5% DMSO. After 4 hours, the cells were sedimented by centrifugation, the medium was aspirated, and then the cell pellet lysed with a 40:40:20 mixture of methanol:acetonitrile:0.5% formic acid in water followed by neutralization with ammonium bicarbonate. Cell debris and precipitates were removed by centrifugation and the supernatants were stored at -80°C until analysis using Waters HPLC-Tandem Mass Spectrometer system.

DHO, N-carbamoyl, and uridine levels in the extract, which were separated by ES-Industry Epi-Polar Column, 5 µm 120Å 10 cm x 4.6 mm, in a mobile phase gradient of 0.1% formic acid in water and 0.1% formic acid in acetonitrile, were detected using a Waters XQ-s Spectrometer. The calibration curve for each metabolite was prepared in the cell lysate solution and included standards from 0.01 to 8 µg/mL.

### AML Patient Primary Cell Analysis

Peripheral blood samples from 10 patients diagnosed with AML were shipped overnight from clinical investigation sites to the test facility. Information on the 10 samples is provided in **Tables 1A** and **1B**. Upon arrival, red blood cells were lysed and the remaining cells counted and resuspended at the appropriate cell concentration in serum free medium supplemented with cytokines (20). The samples were then seeded in 384-well microtiter plates and treated in triplicate with emvododstat or its inactive enantiomer, PTC-371, at concentrations of 32 to 10,000 nM. The samples were also treated as described above with the known DHODH inhibitors brequinar (32 to 10,000 nM) and teriflunomide (317 to 100,000 nM) for comparison. To assess exogenous reversal of emvododstat-mediated inhibition, exogenous uridine (0, 3, 10, 30, or 100 µM) was added to the 384-well microtiter plates for each compound at the time of compound addition.

**Table 1A:** Patient sample information.

Patient Sample	AML42	AML218	AML224	AML237	AML238
Genetics	p53 mutant, NRAS mutant	FLT3 wt, IDH1/2 wt, CEBPα wt	FLT3 ITD; mutNPM1	mutNMP1	ND
Karyotype	Trisomy 8	Normal	ND	ND	ND
Diagnosis	MDS to AML	CMML-2	AML NOS	AML with mutated NPM1	Refractory MDS/AML
Pretreatment	No response to Aza/procrit, currently on hydroxyurea	ND	Naïve	ND	Revlimid, vidaza
% Blasts pretreatment	93	49	95	87	54
Total blasts pretreatment	9379	6046	25279	12879	2049

**Table 1B:** Patient sample information.

Patient Sample	AML293	AML294	AML295	AML299	AML303
Genetics	NMP1 WT/FLT3 WT; KMTA2A normal	NMP1 mut;FLT3 WT	kmt2a rearrangement at 11q23, t(11;19) (q23;p13.1)	MLL-AF9 translocation at t(9;11) (p22;q23)	FLT3 WT;KRAS; 46, XX,t(6;11) (q27;q23)
Karyotype	ND	ND	ND	ND	ND
Diagnosis	AML NOS	AML	AML	Relapsed AML	Secondary AML, treatment-related
Age	18	ND	71	31	57
Sex	M	ND	ND	M	F
CD14 Status	Negative	ND	ND	Positive	Negative
Pretreatment	Naïve	Naïve	ND	3+7, BMT, Relapsed	ND
% Blasts pretreatment	93	77	83	96	79
Total blasts pretreatment	10,693	23,655	14,482	28,855	1,842

After incubation at 37°C for 72 hours, the cells were stained with the appropriate antibodies and evaluated using a flow cytometer. Additional details including the gating strategy are included in the Supplementary section (**Supplementary Figure 1**). Viable cells remaining after each treatment were identified and quantified using cell surface marker expression, cell membrane integrity, and cell morphology to determine the efficacy and selectivity of emvododstat against the blast population. Changes in cell surface marker expression and shifts in morphology indicative of blast differentiation were evaluated for each compound treatment.

### Human Subcutaneous Tumor Xenograft Studies

The day before tumor inoculation, male athymic nude mice were dosed with cyclophosphamide (100 mg/kg) by intraperitoneal injection. Mice were then inoculated in the right flank with 1x10^7^ MOLM-13 AML tumor cells/mouse mixed 1:1 with Matrigel (Corning, AZ, USA). At 7 days post-implantation, mice were randomly divided into groups and administered vehicle (35% Labrasol^®^, 35% Labrafac CC, and 30% Solutol HS 15) or test compounds in vehicle by oral gavage. Tumor volumes were measured twice per week using digital calipers and body weights measured. Tumor volume was determined according to the formula: (Lx(W)^2^)/2, where L is the longest dimension and W is the shortest dimension.

### Pharmacokinetics of Emvododstat Inhibition of DHODH in Rhesus Monkeys

Emvododstat-naïve, non-tumor-bearing rhesus monkeys were dosed by oral gavage with emvododstat (10 mg/kg in a lipid-based formulation). Blood samples were collected prior to dosing and at 0.083, 0.25, 0.5, 1, 2, 4, 6, 8, 12, 16, 24, 32, 48, 72, and 168 hours post-dose. The blood was centrifuged, and the plasma was collected for subsequent analysis using Waters UPLC and TQ-s mass spectrometer.

Protein precipitation with an organic solvent was used to extract testing compounds from plasma before injection onto the column. In a mobile phase gradient of 0.1% formic acid in water and 0.1% formic acid in acetonitrile, emvododstat was separated using a Waters UPLC BEH C18 1.7 µm, 2.1x5.0 mm column and detected by multiple reaction monitoring (MRM) transition 465.6 → 127.3. The biomarkers were separated by ES-Industry Epi-Polar Column, 5 µm 120Å 10 cm x 4.6 mm, and detected by MRM transition 156.95 → 112.95 and 175.1 → 132 for DHO and N-carbamoyl-L-aspartate, respectively. Deuterated (D3)-PTC299 and deuterated (D4)-DHO were used as internal standards for emvododstat and biomarkers analysis, respectively. During sample analysis, each calibration curve spiked with emvododstat in the control plasma was analyzed with the test samples to determine the unknown plasma concentrations. The calibration curve ranged from 0.1 to 8 µg/mL for DHO and 0.002 to 2 µg/mL for emvododstat.

## Results

### Emvododstat Shows Broad Activity Against Leukemia/Lymphoma Cell Lines

Previously, Cao etal. (13) showed emvododstat (PTC299) has broad and potent activity against 240 different hematologic cancer cell lines, with leukemia/lymphoma cell lines being more sensitive than solid tumor lines (13). To assess further the mechanism of action underlying the effect of emvododstat, a subset of leukemia cell lines with varying degrees of sensitivity were retested for emvododstat sensitivity and subsequently used in metabolomic studies. Proliferation of 7 of the 12 cell lines tested showed high sensitivity to emvododstat (IC_50_ <30 nM) (**Table 2**). Four cell lines were insensitive (TF-1, Sup-B15, RS4;11, THP-1 [IC_50_ ≥4000 nM]). The cell line HL60 showed moderate sensitivity to emvododstat (IC_50_ = 592.5 nM).

**Table 2:** Sensitivity of leukemia cell line proliferation to emvododstat.

Cell Line	Description	Driver Mutation	IC_50_ (nM)
MOLM-13	AML heterozygous for the FLT3-ITD	FTL3-ITD (21)	7.17
Molt4	T-cell ALL	NOTCH1 (22) NRAS (23) PTEN (23) TP53 (23)	5.8
U937	Pro-monocytic, AML	PTEN (24) TP53 (25)	8
Jurkat	T-cell ALL	BAX (26) NOTCH1 (22) TP53 (27)	9.4
K562	Erythroleukemia	BCR-ABL1 (28) TP53 (29)	11.5
MV4-11	AML homozygous for the FLT3-ITD	FTL3-ITD (30)	27
Sup-T1	T-cell lymphoblastic lymphoma	EGFR (27) TP53 (27)	28.1
HL60	APL	NRAS (23) TP53 (31)	592.5
TF-1	Erythroleukemia	TP53 (25)	≥4000
Sup-B15	B cell precursor ALL	BCR-ABL1 (32)	≥4000
RS4;11	ALL that exhibits B lineage and monocytic characteristics	KMT2A-AFF1 (33)	≥4000
THP-1	Acute monocytic leukemia	CDKN2A, CDKN2B, PTEN (34), TP73, MLL-AF9 fusion	≥4000

Cells were treated with increasing concentrations of emvododstat (4.57 to 10,000 nM) and were subsequently evaluated for viability after 72 hours using CellTiter-Glo Luminescent Cell Viability Assay kit. The IC_50_ (concentration resulting in 50% reduction in ATP levels) was determined.

### Evaluation of Baseline Levels and Emvododstat-Induced Changes in the Levels of DHODH Substrates and the Salvage Metabolite Uridine

**Figure 2:**
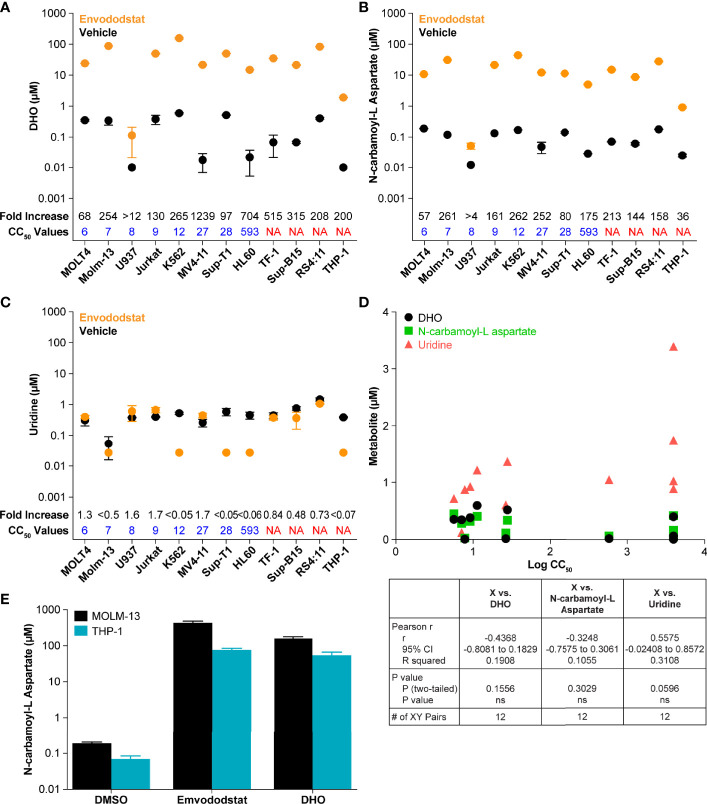
The figures show baseline (untreated) and post-treatment levels for the metabolites DHO **(A)** and N-carbamoyl-L-aspartate **(B)**, and uridine **(C)** following treatment with 1 µM emvododstat. Included for each cell line is the CC_50_ just above the x-axis and the fold increase (treated/untreated) measured in each cell line at the top of the graph. Values represent the mean ± SD for 3 replicates. **(D)** Shown are the baseline levels of DHO, N-carbamoyl-L-aspartate, and uridine vs the log CC_50_ values. Below the graph are the correlation statistics calculated using Prism (GraphPad). **(E)** Shown are levels of N-carbamoyl-L-aspartate measured in MOLM-13 or THP1 cells after 4 h of incubation with 1 µM emvododstat or with 10 mM DHO. Where levels of a measured metabolite were below the lower limit of quantification (LLOQ), a value of LLOQ/2 was used for subsequent calculations.

To further demonstrate that emvododstat is acting *via* the *de novo* pyrimidine nucleotide biosynthesis pathway in the different cell lines, the levels of DHO, the substrate of DHODH, as well as the levels of N-carbamoyl-L-aspartate, the metabolite one step above DHODH (see **Figure 1**), were measured across the 12 cell lines. Consistent with inhibition of DHODH by emvododstat, the levels of DHO and N-carbamoyl-L-aspartate increased in all 12 cell lines (**Figure 2**). Interestingly, no correlation was established between IC_50_ and baseline levels of DHODH substrates (shown as black circles in **Figures 2A, B, D**), levels of DHODH substrates after treatment (shown as orange circles), or changes in the DHODH substrates (shown as fold increase above values in **Figures 2A, B**). This indicates that the lack of sensitivity to emvododstat is not due to the inability of emvododstat to inhibit the enzyme activity. The levels of uridine, a component of the salvage pathway, was also evaluated. Interestingly, as shown in **Figure 2D**, baseline levels of uridine did correlate with sensitivity (although the correlation did not quite reach statistical significance) suggesting that cell lines that were less sensitive may have higher levels of metabolites attributed to the salvage pathway. These data demonstrate that DHODH is inhibited in each leukemia/lymphoma cell line, but that not all of these cell lines are reliant on *de novo* pyrimidine nucleotide synthesis for survival and may instead effectively use the salvage pathway for proliferation.

The increase in DHO was expected based on previous studies (13) and increases in DHO seen in DHODH-deficient patients (35). However, the increase in the levels of N-carbamoyl-L-aspartate was not expected. This was further evaluated using MOLM-13 cells (proliferation reduced by emvododstat with a IC_50_ of 5 nM) and THP-1 cells (proliferation not affected by emvododstat, IC_50_ >4000 nM). As shown in **Figure 2E**, the addition of emvododstat increased levels of N-carbamoyl-L-aspartate, consistent with **Figure 2B**. The addition of exogenous DHO also increased the levels of N-carbamoyl-L-aspartate, indicating that DHO may inhibit the carbamoyl-phosphate synthetase 2 enzyme (**Figure 1**).

### Activity of Emvododstat Against Primary AML Samples From Patients

To confirm that the activity seen against cell lines was relevant to AML, studies were performed using primary AML blasts in whole blood samples of AML patients cultured ex vivo. Information on the genetics, karyotype, diagnosis, and pretreatment of each patient is shown in **Table 1A**. The impact of emvododstat on cell viability was assessed by measuring total and immature blast cell numbers.

**Figure 3:**
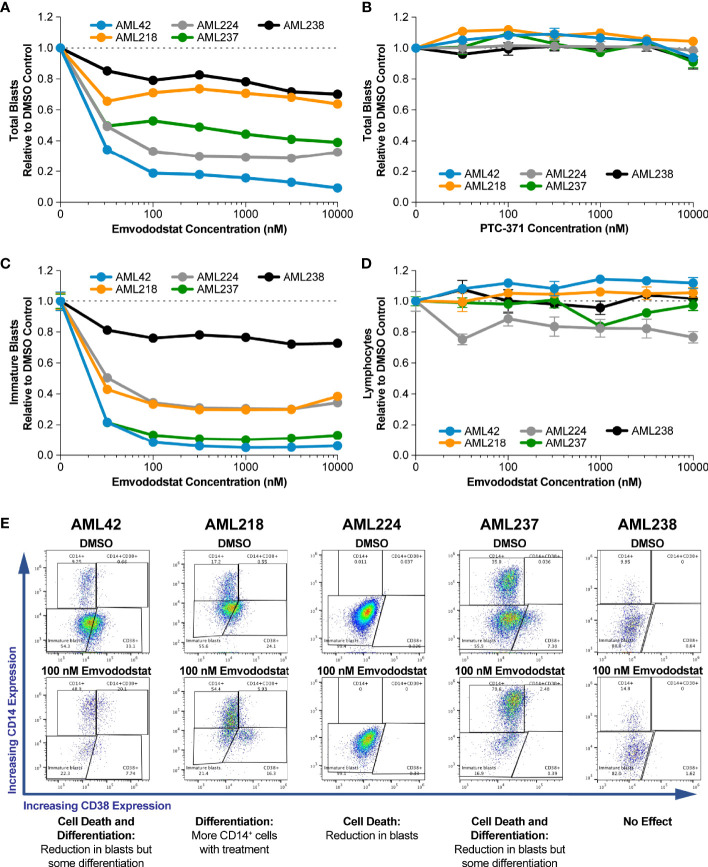
Primary AML blasts cultured ex vivo were treated with increasing concentrations of emvododstat or its inactive enantiomer PTC-371 at 37°C for 72 hours. Samples were then stained with appropriate antibodies and evaluated using a flow cytometer. For panels **(A-D)**, values represent the mean ± SD for triplicate values. Decrease in the fraction relative to control of **(A)** total blast cells treated with emvodostat, **(B)** total blast cells treated with PTC-371, **(C)** immature blast cells treated with emvodostat, or **(D)** lymphocytes treated with emvodostat. **(E)** Flow cytometry analysis of primary AML patient samples. Shown are data from blood obtained from 5 AML subjects incubated ex vivo with 100 nM emvodostat or DMSO.

Emvododstat is chiral and only the (*S*)-enantiomer is active (13). PTC-371, the inactive (*R*)-enantiomer, did not affect cell viability, indicating the effect was emvododstat specific (**Figures 3A, B**). Data are shown normalized to vehicle control (DMSO) blast number. **Supplementary Figure 2** and **Supplementary Table 1** shows absolute total blast numbers. Emvododstat elicited at least a 20% cell reduction at concentrations ≥32 nM in primary AML samples from 4 of the 5 subjects (**Figures 3C, D**). At a concentration of 100 nM, emvododstat-dependent reduction in total blasts ranged from 21% to 81% and reductions in immature blasts ranged from 24% to 91%. Reductions in immature blast cells accounted for most of the loss in total blasts cells (**Supplementary Figure 3**). For sample AML218, the reduction in immature blast cells was accompanied by an increase in differentiated (CD14+) blasts cells without a reduction in the total blast number. **Supplementary Table 2** shows the percentage of blasts relative to total live numbers before and after treatment. Because most live cells evaluated are blasts, this number does not change must pre- vs post-treatment.

**Figure 3E** shows data for the 5 primary AML samples incubated with 100 nM emvododstat. The y-axis of each graph shows the percentage of cells positive for CD14 staining and the x-axis of each graph shows the percentage of cells positive for CD38 staining. Increased levels of CD14 staining are associated with a more differentiated myeloid phenotype. Each of the 5 patients responded differently, showing varying levels of decreases in total blasts and increases in CD14 staining. For patients AML42 and AML237, emvododstat resulted in a reduction in the CD14-negative blast population while increasing the percentage of CD14-positive cells. Emovdodstat had no effect on the AML238 blasts. Subject AML238 had the lowest total blast percentage on Day 0 (**Table 1A**).

### Emvododstat Promotes Myeloid Blast Cell Differentiation

Prior studies suggested that inhibition of DHODH may not only result in loss of myeloid viability but also promotes myeloid differentiation (36). Therefore, the ability of emvododstat to promote differentiation of primary AML blasts was evaluated. Toward this end, 5 additional blood samples from AML subjects (see **Table 1B**) were obtained and cultured ex vivo as previously described. The number of AML blast cells expressing CD14, a marker associated with myeloid differentiation, following treatment with emvododstat or control was evaluated by flow cytometry. Increased differentiation was defined as ≥30% increase in blast CD14+ cells.

**Figure 4:**
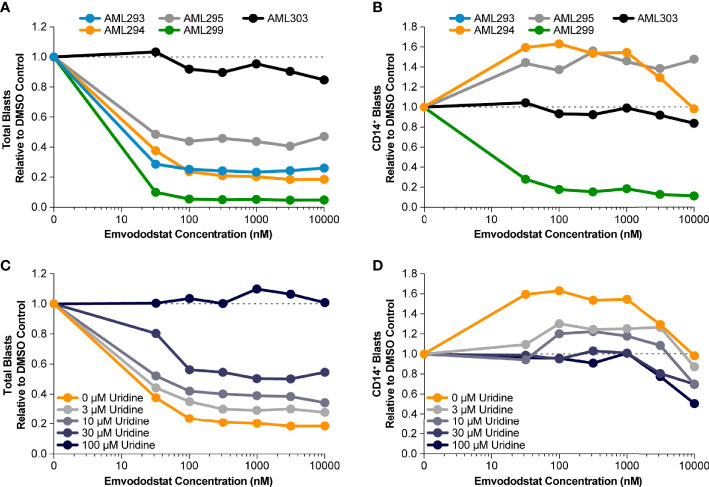
Primary AML blasts cultured ex vivo were treated with increasing concentrations of emvododstat at 37°C for 72 hours. Then samples were stained with appropriate antibodies and evaluated using a flow cytometer. Values represent the mean ± SD for triplicate values. Shown is the decrease in the fraction relative to control of **(A)** total blast cells, **(B)** CD14+ treated with increasing concentration of emvododstat in primary cell cultures from 5 patients with AML. **(C)** Using sample AML294, addition of ≥30 µM of uridine reversal of the effect of emvododstat on reduction in total blasts. **(D)** Using sample AML294, addition of ≥30 µM of uridine reversed the effect of emvododstat on the increase in CD14+ cells.

The fraction total blasts cells relative to vehicle decreased in 4 of the 5 samples (**Figure 4A**). As summarized in **Supplementary Table 1**, because the samples contained predominately blasts, the percentage of blasts relative to total live cells did not change or decreased with emvododstat treatment. Data are shown normalized to vehicle control (DMSO) blast number; **Supplementary Figure 2** shows absolute total blast number. Subject AML303 had the lowest total blast percentage at Day 0 (**Table 1B**). At the concentrations of emvododstat tested, an increase of 36% to 96% of CD14+ cells was observed in 2 of the 5 previously described patient AML samples (**Figure 4B**). At higher concentrations, a decrease in the percentage of CD14+ cells was observed in these 2 samples, indicating that at higher concentrations cytotoxicity and not differentiation is induced.

To evaluate if emvododstat promotes cytotoxicity and differentiation *via* inhibition of DHODH, the ability of exogenously added uridine to rescue cell viability was tested using the sample from subject AML294. Addition of uridine bypasses the *de novo* pyrimidine synthesis pathway (see **Figure 1**). In the absence of uridine, emvododstat reduced the total percentage of blasts. As expected for a DHODH inhibitor, uridine reversed both the cytotoxic and differentiation effects of emvododstat. Uridine dose-dependently prevented the emvododstat-induced reduction in total blasts (**Figure 4C**). Uridine also dose-dependently blocked myeloid differentiation (**Figure 4D**). Similar data are shown for subject AML293 and AML95 as well as for MOLM13 cells (**Supplementary Figure 4**).

Exogenously added uridine in a concentration-dependent manner reversed the emvododstat-induced myeloid blast cell death in all 5 AML patient-derived primary cell samples (**Figure 4C** and data not shown) in a concentration-dependent manner. These results are consistent with emvododstat acting by inhibiting DHODH and not by promoting off-target, nonselective cytotoxicity.

### Potency of Emvododstat Compared With Brequinar and Teriflunomide

The potency of emvododstat to promote cytotoxicity and differentiation of myeloid cells relative to that of the DHODH inhibitors brequinar and teriflunomide was evaluated using the primary cell cultures derived from the second set of 5 AML subjects (AML293, AML294, AML295, AML299, and AML303; see **Table 1B**). These AML patient samples demonstrated greater sensitivity to emvododstat than to brequinar or teriflunomide. In 4 of the 5 cultured patient samples, the IC_50_ for emvododstat was ≤31 nM compared with ≥219 nM for brequinar and ≥7280 nM for teriflunomide (**Table 3**). Cells in the blood sample derived from Patient AML303 were not responsive to any of the 3 compounds.

**Table 3:** Summary of sensitivity of primary AML cell culture with emvododstat, brequinar, and teriflunomide.

Compound	Patient Number				
	AML293	AML294	AML295	AML299	AML303
Emvododstat, CC_50_ (nM)	22	26	31	18	>10,000
Brequinar, CC_50_ (nM)	219	397	23,682	251	>10,000
Teriflunomide, CC_50_ (nM)	7,280	>10,000	12,439	7,378	>10,000

Each patient sample was treated for 72 hours with DMSO, emvododstat (32 to 10,000 nM), brequinar (32 to 10,000 nM), or teriflunomide (32 to 10,000 nM). The concentration to reduce blast number by 50% (CC_50_) was calculated by assuming the percent inhibition increased linearly from 0% to 50%.

### Emvododstat Monotherapy Shows Activity in a MOLM-13 AML Xenograft Mouse Model

To assess the physiological relevance of the *in vitro* effects of emvododstat, studies were done to assess inhibition of the DHODH by emvododstat *in vivo*. Studies were performed in a MOLM-13 AML xenograft mouse models to correlate inhibition of DHODH with increases in DHO levels and reduction in tumor growth. In this model, MOLM-13 AML cells were grown as a solid tumor in immunocompromised mice. Previously, we have shown that emvododstat (PTC299) does not inhibit murine DHODH (13), and consequently, any effect of the drug on DHO levels and tumor growth would be a direct effect of emvododstat on the human DHODH in the xenograft itself.

**Figure 5:**
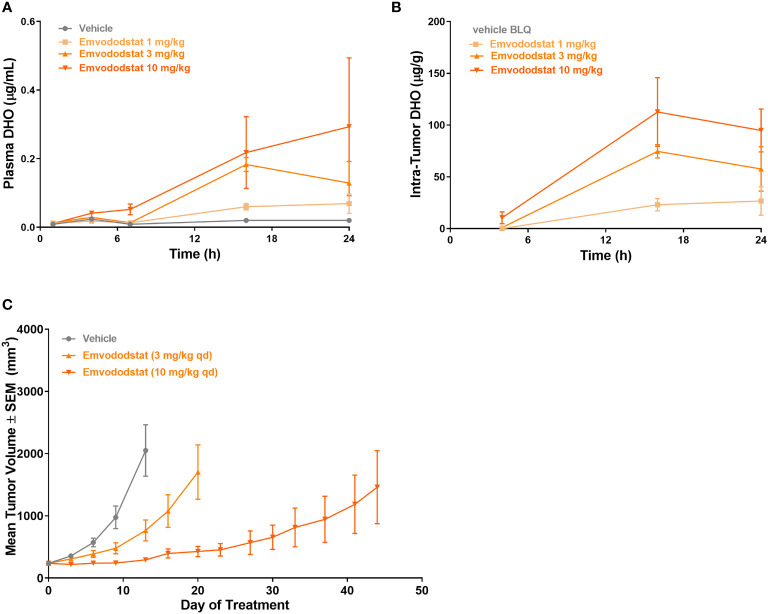
Mice bearing MOLM-13 tumors were dosed with emvododstat and at each timepoint, 3 mice per group were euthanized. **(A)** plasma and **(B)** tumors in a mouse xenograft model. **(C)** MOLM-13 tumor growth. Values represent the mean ± SEM of 10 mice/group.

Mice bearing MOLM-13 tumors (425 ± 213 cm^3^) were dosed with emvododstat and euthanized at specified timepoints through 24 hours post-dose. Dose-dependent increases in plasma and tumor DHO levels were observed following emvododstat treatment but not with vehicle control (**Figures 5A, B**). In a second study using the same doses of emvododstat, there was a dose-dependent reduction in the rate of tumor growth compared with vehicle control (**Figure 5C**). The inhibition of tumor growth by emvododstat was more prolonged at 10 mg/kg compared with 3 mg/kg despite both doses resulting in the inhibition of DHODH as shown by the increased levels of DHO. However, the more sustained inhibition of DHODH with the higher drug dose suggests that greater efficacy is associated with sustained inhibition of the DHODH.

To confirm further the effect of DHODH inhibition *in vivo*, a second study was performed with the structurally related DHODH inhibitor, PTC-868 (37). As shown in **Supplementary Figure 5A**, PTC-868 reduced the rate of MOLM-13 tumor growth. At the end of the study, the mean tumor volume from mice dosed with PTC-868 was 1296 cm^3^ (larger than those in **Figures 5A, B**). Levels of DHO and N-carbamoyl-L-aspartate were measured in plasma from mice dosed with vehicle or with PTC-868 (**Supplementary Figure 5B**). In mice dosed with vehicle, plasma levels of both DHO and N-carbamoyl-L-aspartate were below the lower limit of quantification. Consistent with increases in DHO substrates measured *in vitro*, the levels of DHO and N-carbamoyl-L-aspartate were increased in plasma from mice dosed with PTC-868.

### Emvododstat Inhibits DHODH in Primates

To examine further the effectiveness of emvododstat inhibition of DHODH *in vivo*, the effect of the drug in rhesus monkeys was assessed. Studies using green monkey COS cells showed that emvododstat inhibited monkey DHODH (data not shown).

Following a single 10 mg/kg oral dose in drug naïve, non-tumor-bearing non-human primates (NHPs), emvododstat plasma concentrations increased with a T_max_ of 2 hours (**Supplementary Figure 6**). Levels of the drug subsequently decreased, but were measurable through 72 hours, with a terminal half-life of approximately 33 hours. Consistent with inhibition of DHODH by emvododstat, the levels of the DHODH substrate, DHO, which were below the lower limit of quantification (0.05 µg/mL) before treatment, rose after about 2 hours indicating that the inhibition of DHODH is rapid. The peak increase in DHO (C_max_ 2.48 µg/mL) occurred at 24 hours. DHO levels subsequently declined but were detectable through 72 hours, with a half-life of approximately 10 hours, indicating that inhibition of DHODH is reversible.

Relative levels of DHO were much higher in the NHP than those observed in the mouse xenograft studies consistent with the species selectivity of emvododstat. In the xenograft studies, the measured DHO represents DHO from the human xenograft only. In the non-tumor-bearing NHP, the DHO is from normal somatic tissue as all tissues express DHODH.

## Discussion

Inhibition of the *de novo* pyrimidine nucleotide synthesis pathway has been associated with blast cell differentiation, inhibition of proliferation, and cell death in AML cells (17, 18). Rapidly proliferating cells, such as leukemia/lymphoma blasts, including AML, show a selective vulnerability to DHODH blockade (14, 15). Results from the study described here show that emvodostat demonstrates a dual mode of action against AML, inhibiting proliferation and inducing cell differentiation. In this study, we demonstrated that multiple leukemia cell lines were sensitive to emvododstat. Of those showing sensitivity, the IC_50_ values ranged from 5.8 to 28.1 nM versus ≥4000 nM for the resistant cell lines. Differences in sensitivity to emvododstat did not relate to differences in the sensitivity of the enzyme to inhibition; emvodostat inhibited DHODH in all 12 cell lines tested as shown by the accumulation of substrate. The differences in sensitivity likely reflect the differences in the reliance of the AML cells upon *de novo* vs salvage for pyrimidine nucleotides. Consistent with this hypothesis, baseline levels of the salvage metabolite uridine correlated with sensitivity of cells to emvododstat. Cell lines with high baseline uridine levels were generally less sensitive. In support of this, we previously reported that cells with lower salvage uridine monophosphate (UMP) production relative to *de novo* UMP production were more sensitive to emvododstat than those with higher salvage activity (13).

Emvododstat demonstrated activity against AML blast cells from AML patient blood cultured ex vivo; at a concentration of 100 nM, total blasts were reduced between 21% to 81% and immature blasts were reduced between 24% to 91%. Emvododstat also promoted myeloid differentiation as determined by the increase in the fraction of CD14+ cells. Since multiple cell lines were sensitive, emvododstat may be effective against a series of AML subtypes and highlights the importance of the *de novo* pyrimidine biosynthesis pathway as a regulator of myeloid blast cell survival and differentiation. Interestingly, emvododstat induced differentiation in some primary AML cell lines, a reduction in blast number in others, and both differentiation and a reduction in blast number in others. This variability across different primary AML samples likely reflects differences in the underlying driver mutations resulting in some cells relying greater upon the *de novo* pyrimidine biosynthesis pathway than on the salvage pathway. The addition of exogenous uridine blocked the emvododstat effect on cell death and differentiation consistent with emvododstat acting *via* DHODH to block cell proliferation and enhance blast cell differentiation.

The increase in levels of N-carbamoyl-L-aspartate, which is upstream of DHO and DHODH in the pathway, suggests emvododstat influences a feedback loop that controls the activity of other enzymes in the pathway. The CAD complex (carbamoyl-phosphate synthetase 2, aspartate carbamoyl transferase, and dihydroorotase) has been shown to be a site of allosteric regulation and feedback (38, 39).

DHO levels were also shown to increase *in vivo* after emvododstat administration demonstrating that emvododstat targets DHODH *in vivo*. Emvododstat inhibited tumor growth of MOLM-13 AML cells in a murine xenograft model. Furthermore, an association was observed between increasing doses of emvododstat and increasing levels of both plasma and tumor DHO. In the NHP, emvododstat plasma concentration rapidly increased within 2 hours of dosing. The time for the peak level of DHO, the substrate for DHODH, was delayed relative to the maximal emvododstat concentration. These data suggest that clinical dosing regimens that result in sustained increases in drug levels and sustained inhibition of DHODH, as measured by increased DHO levels *in vivo*, will be more effective in AML treatment.

In this study, emvododstat was found to be more potent in promoting cell death than the DHODH inhibitors, brequinar or teriflunomide, in primary AML blasts derived from AML patient blood samples. Several other inhibitors of DHODH have been investigated for the treatment of cancer, these include brequinar, leflunomide, its active metabolite teriflunomide, IMU-383 (vidofludimus), BAY 2402234, and ASLAN003 (18, 36, 40–44). Currently, brequinar is not approved for use in any indication (42, 45, 46). ASLAN003 was previously found to be considerably less potent than emvododstat in MOLM-13 AML cells (CC_50_ 152 nM vs 3 nM, respectively) (18).

The selective inhibition of the DHODH enzyme with emvododstat is not associated with cytotoxicity of lymphocytes (see **Figure 3D**) and was shown to be safe in investigational new drug (IND)-enabling toxicology studies (13). This reflects that these normal differentiated cells obtain pyrimidine nucleotides *via* salvage pathways and is consistent with the absence of cytopenia, as observed with emvododstat in IND-enabling toxicology studies and in the clinic (19, 47, 48).

In summary, emvododstat inhibition of DHODH shows a dual mode of action in targeting AML: (1) inhibition of cell proliferation; and/or (2) promotion of differentiation. The dual effect of emvododstat arises from inhibition of the *de novo* synthesis pathway and pyrimidine nucleotide depletion, as shown by the ability of exogenous uridine to reverse the effects of emvododstat. The selectivity of emvododstat on rapidly proliferating cells contributes to its potential for efficacy in AML in the absence of general toxicity. Because emvododstat is effective in multiple AML models that represent different AML genotypes, it may have broad therapeutic applicability in myeloid malignancies. Emvododstat has been extensively studied in the clinic and has favorable pharmacokinetic and safety profiles. Currently, the therapeutic potential of emvododstat is being explored in an ongoing open-label Phase 1 study of patients with relapsed/refractory AML (NCT03761069). These data, together with previous extensive biological characterization of emvododstat (PTC299) (13, 19), provide evidence for emvododstat as a potent and selective monotherapy, with broad potential applicability across genetically diverse subtypes of AML, and provide a strong rationale for further clinical evaluation of this drug in treating leukemias.

## Data Availability Statement

The raw data supporting the conclusions of this article will be made available by the authors, without undue reservation.

## Ethics Statement

Studies using mice were done at Rutgers University, an AAALAC certified facility, and done under Rutger’s IACUC-approved protocols. The PK/PD study in the non-human primates was done at Covance laboratory (Madison, WI), an AAALAC certified facility, and done under Covance IACUC-approved protocols.

## Author Contributions

Conceptualization, data acquisition and curation, formal analysis, methodology, and project administration: AB, LC, BF, CT, JG, JC, BR, WL, JS, AM, MS, SY, RK, RSp, JB, KO’K, and MW. Writing, review, and editing: AB, LC, BF, CT, JG, JC, BR, WL, JS, AM, MS, SY, RK, JB, KO’K, RSp, EG, RSh, SK, and MW. All authors contributed to the article and approved the submitted version.

## Funding

This study was funded by PTC Therapeutics, Inc.

## Conflict of Interest

Authors AB, LC, BF, CT, JG, JM, BR, WL, JS, AM, SY, RK, JB, KO’K, RSp, EG, SK and MW are or were employed by PTC Therapeutics and have received salary compensation for time, effort, and hold or held financial interest in the company. Author MS was employed by Notable Labs and RSh was employed by InSeption Group.

The authors declare that this study received funding from PTC Therapeutics, Inc. The funder had the following involvement with the study: funded the entire study, including all experiments and outside editorial support.

## Publisher’s Note

All claims expressed in this article are solely those of the authors and do not necessarily represent those of their affiliated organizations, or those of the publisher, the editors and the reviewers. Any product that may be evaluated in this article, or claim that may be made by its manufacturer, is not guaranteed or endorsed by the publisher.

## Supplementary Material

Supplementary Figure 1 Using the gating strategy shown in the table and the figure, viable cells remaining after each treatment were identified and quantified using cell surface marker expression, cell membrane integrity, and cell morphology to determine the efficacy and selectivity of emvododstat against the blast population. Changes in cell surface marker expression and shifts in morphology indicative of blast differentiation were also evaluated for each compound.

Supplementary Figure 2 Primary AML blasts cultured ex vivo were treated withincreasing concentrations of emvododstat at 37°C for 72 hours. Samples were thenstained with appropriate antibodies and evaluated using a flow cytometer. Valuesrepresent the mean ± SD for triplicate values of absolute numbers of blasts. **(A)** Absolute number of blasts for primary blast samples shown in **Figure 3**. **(B)** Absolute number of blasts for primary blasts samples shown in **Figure 4**.

Supplementary Figure 3 Primary AML blasts cultured ex vivo were treated with increasing concentrations of emvododstat. Blasts cells were identified and further defined as differentiated (CD14+) or undifferentiated.

Supplementary Figure 4 Uridine reverses emvododstat-mediated effects ontotal blast counts in primary AML blasts and on AML cell lines. **(A)** total blasts cellsfrom sample AML293, addition of ≥30 μM of uridine reversal of the effect ofemvododstat on reduction in total blasts; **(B)** total blasts cells from sample AML299,addition of ≥30 µM of uridine reduces the effect of emvododstat on reduction in totalblasts, with complete reversal at 100 µM. **(B)** AML303 does not respond toemvododstat. **(C)** Uridine rescue has no effect on doxorubicin-induced cytotoxicity of MOLM-13. **(D)** Uridine has no effect on viability of blocksMOLM13 cells (as measuredusing CTG). Uridine rescues emvododstat-induced inhibition of MOLM-13 cell viability; addition of ≥30 μM of uridine reduces the effect of emvododstat on reduction in total blasts,with complete reversal at 100 µM.

Supplementary Figure 5 Mice bearing MOLM-13 tumors were dosed with PTC-868 and at each timepoint, 3 mice per group were euthanized. Values represent the mean ± SEM. **(A)** Inhibition of MOLM 13 tumor growth with treatment of PTC-868 (1 mg/kg, PO). Values represent the mean ± SEM for N=10 mice/group. **(B)** Increased DHO and N-carbamoyl-L-aspartate in plasma obtained on Day 12 in the study shown in panel A. Levels in vehicle control were below the lower limit of quantification.

Supplementary Figure 6 After a single dose of emvododstat, blood was obtained at specified timepoints and processed to plasma. Plasma was analyzed for levels of emvododstat, DHO, and uridine. The blue line shows levels emvododstat, the orange line shows the levels of DHO.
