# Supplementary figures and images for: Emvododstat, a Potent Dihydroorotate Dehydrogenase Inhibitor, Is Effective in Preclinical Models of Acute Myeloid Leukemia
Source: Front Oncol. 2022 Feb 9;12:832816. doi: 10.3389/fonc.2022.832816 (PMC8864546; doi:10.3389/fonc.2022.832816)

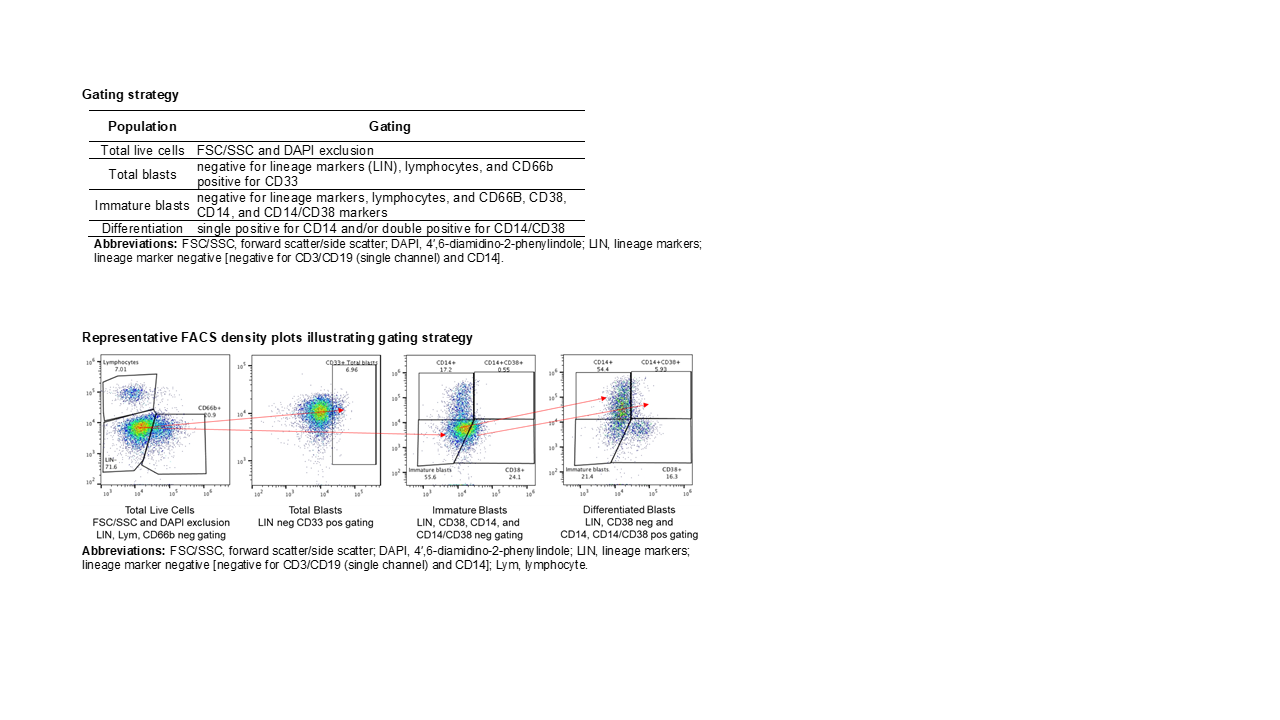

Supplement: Supplementary Figure 1 — Using the gating strategy shown in the table and the figure, viable cells remaining after each treatment were identified and quantified using cell surface marker expression, cell membrane integrity, and cell morphology to determine the efficacy and selectivity of emvododstat against the blast population. Changes in cell surface marker expression and shifts in morphology indicative of blast differentiation were also evaluated for each compound. [file Image_1.tif]

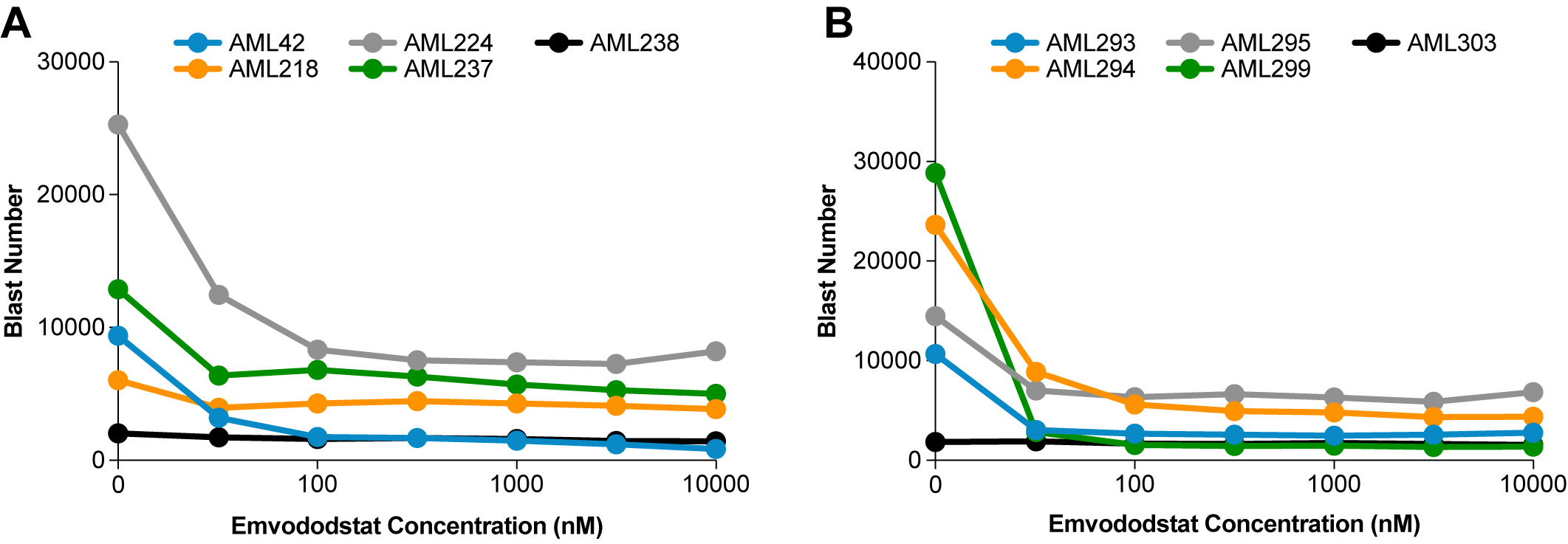

Supplement: Supplementary Figure 2 — Primary AML blasts cultured ex vivo were treated withincreasing concentrations of emvododstat at 37°C for 72 hours. Samples were thenstained with appropriate antibodies and evaluated using a flow cytometer. Valuesrepresent the mean ± SD for triplicate values of absolute numbers of blasts. (A) Absolute number of blasts for primary blast samples shown in Figure 3 . (B) Absolute number of blasts for primary blasts samples shown in Figure 4 . [file Image_2.tif]

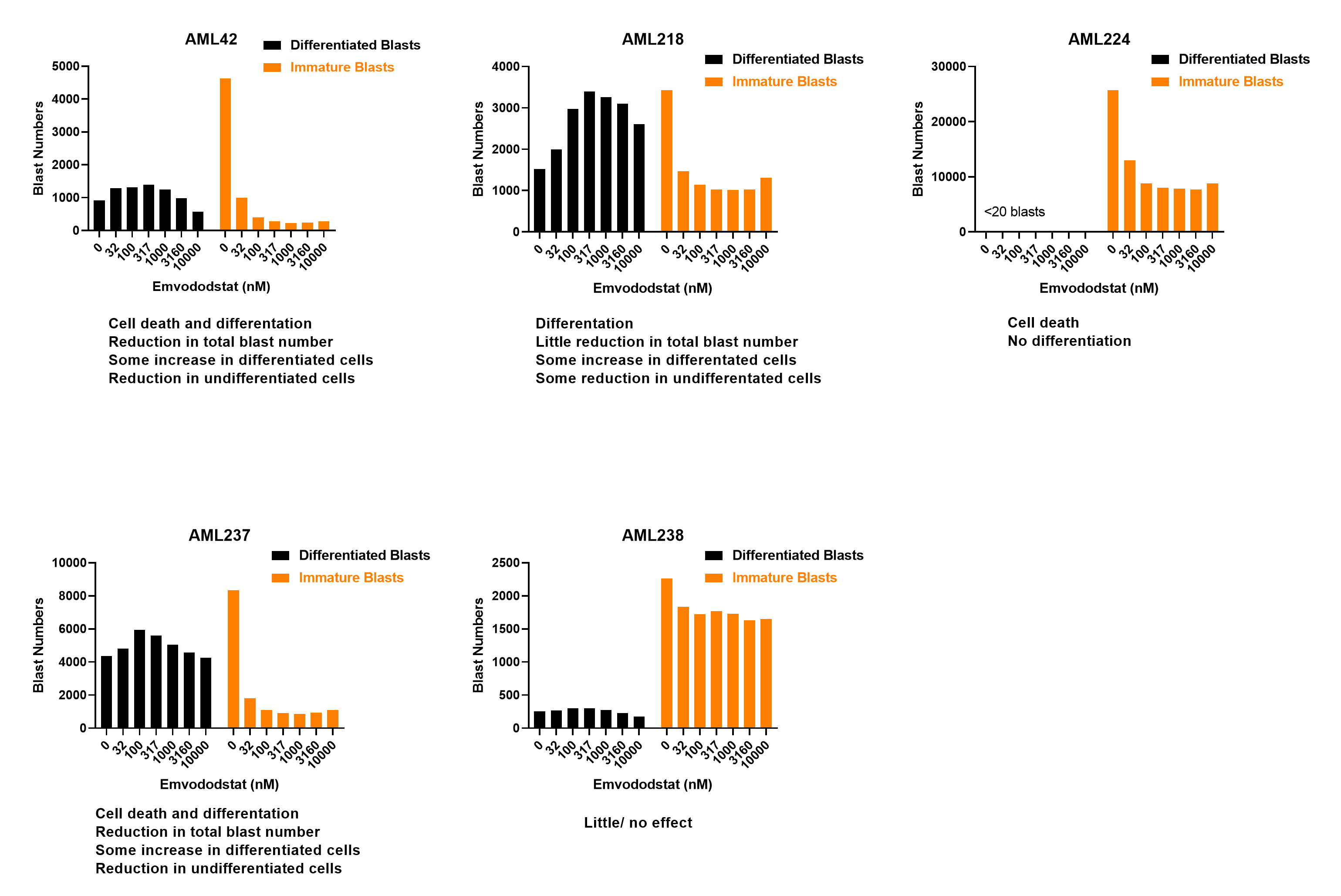

Supplement: Supplementary Figure 3 — Primary AML blasts cultured ex vivo were treated with increasing concentrations of emvododstat. Blasts cells were identified and further defined as differentiated (CD14+) or undifferentiated. [file Image_3.tif]

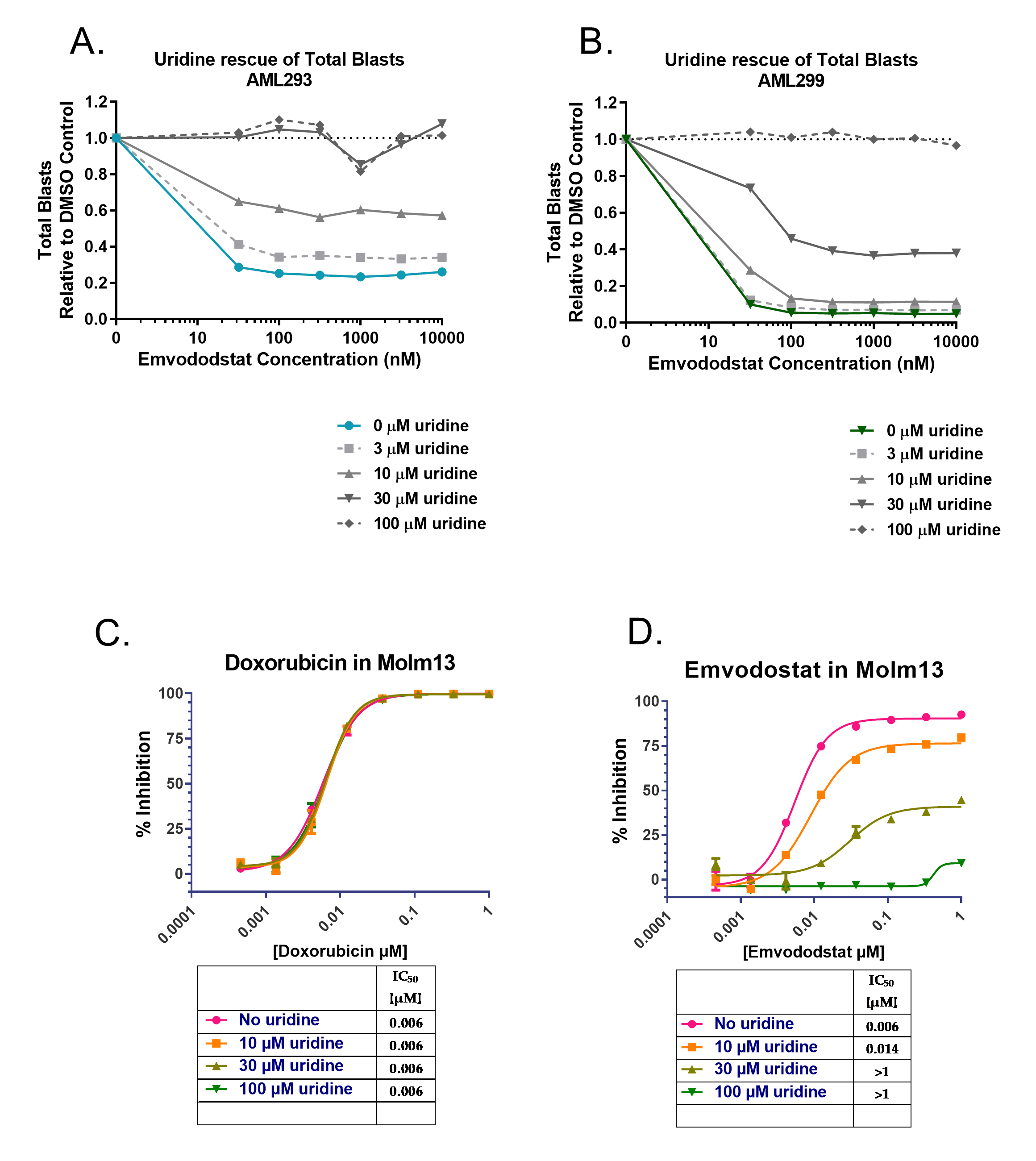

Supplement: Supplementary Figure 4 — Uridine reverses emvododstat-mediated effects ontotal blast counts in primary AML blasts and on AML cell lines. (A) total blasts cellsfrom sample AML293, addition of ≥30 μM of uridine reversal of the effect ofemvododstat on reduction in total blasts; (B) total blasts cells from sample AML299,addition of ≥30 µM of uridine reduces the effect of emvododstat on reduction in totalblasts, with complete reversal at 100 µM. (B) AML303 does not respond toemvododstat. (C) Uridine rescue has no effect on doxorubicin-induced cytotoxicity of MOLM-13. (D) Uridine has no effect on viability of blocksMOLM13 cells (as measuredusing CTG). Uridine rescues emvododstat-induced inhibition of MOLM-13 cell viability; addition of ≥30 μM of uridine reduces the effect of emvododstat on reduction in total blasts,with complete reversal at 100 µM. [file Image_4.tif]

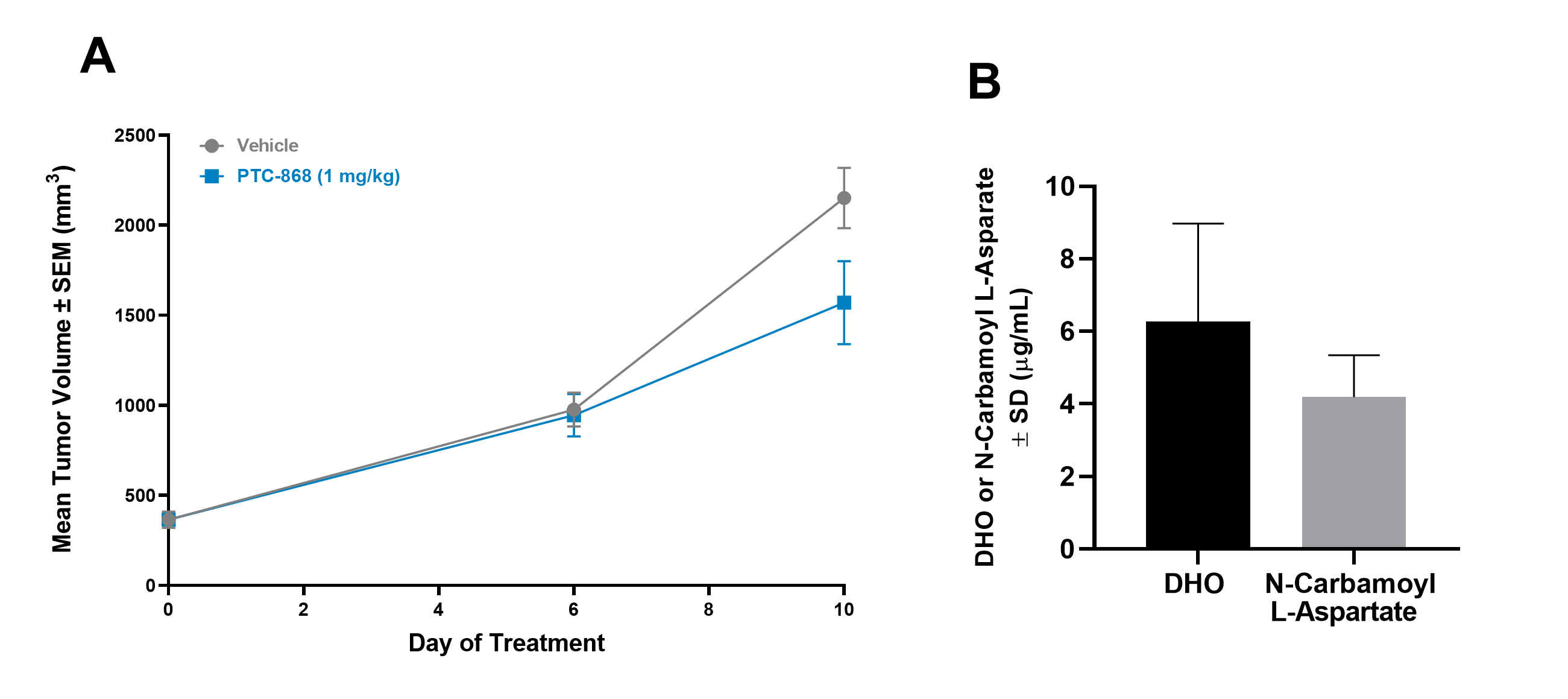

Supplement: Supplementary Figure 5 — Mice bearing MOLM-13 tumors were dosed with PTC-868 and at each timepoint, 3 mice per group were euthanized. Values represent the mean ± SEM. (A) Inhibition of MOLM 13 tumor growth with treatment of PTC-868 (1 mg/kg, PO). Values represent the mean ± SEM for N=10 mice/group. (B) Increased DHO and N-carbamoyl-L-aspartate in plasma obtained on Day 12 in the study shown in panel A. Levels in vehicle control were below the lower limit of quantification. [file Image_5.tif]

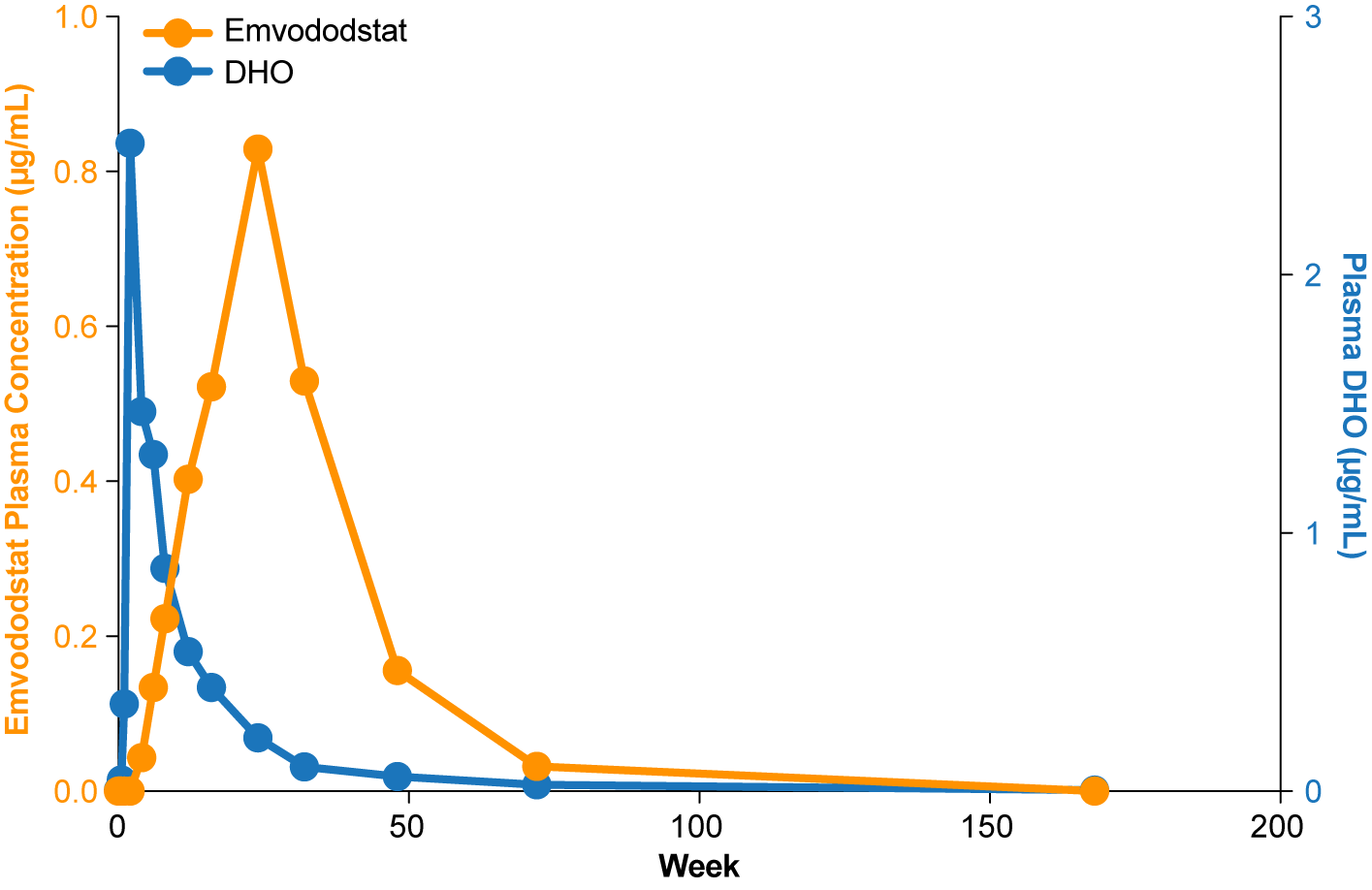

Supplement: Supplementary Figure 6 — After a single dose of emvododstat, blood was obtained at specified timepoints and processed to plasma. Plasma was analyzed for levels of emvododstat, DHO, and uridine. The blue line shows levels emvododstat, the orange line shows the levels of DHO. [file Image_6.tif]
