# Supplementary material for: Emvododstat, a Potent Dihydroorotate Dehydrogenase Inhibitor, Is Effective in Preclinical Models of Acute Myeloid Leukemia
Source: Front Oncol. 2022 Feb 9;12:832816. doi: 10.3389/fonc.2022.832816 (PMC8864546; doi:10.3389/fonc.2022.832816)
Supplement: Supplementary file 7 [file Table_1.docx]

**Supplementary Table 1. Changes in total live cells, total blasts, immature blasts, and biomarkers with emvododstat treatment**

| **Emvododstat concentration** | **CD66b+** | **lymphocytes** | **CD14+ CD38+** | **CD14+**  **CD38-** | **CD14 TOTAL** | **CD38+ CD14-** | **CD34+ KIT+** | **CD34+** | **Kit+** | **Total blasts** | **Immature blasts** | **Total live cells** | **LIN-** |
| --- | --- | --- | --- | --- | --- | --- | --- | --- | --- | --- | --- | --- | --- |
|  | **PATIENT AML42 AVERAGE COUNTS** | | | | | | | | | | | | |
| DMSO (0 nM) | 218 | 268 | 72 | 842 | 914 | 3839 | 71 | 6051 | 117 | 9379 | 4630 | 10112 | 9598 |
| 32 nM | 179 | 290 | 198 | 1084 | 1282 | 1057 | 39 | 1431 | 64 | 3209 | 1002 | 3901 | 3404 |
| 100 nM | 190 | 300 | 268 | 1044 | 1312 | 143 | 8 | 483 | 11 | 1767 | 394 | 2389 | 1882 |
| 317 nM | 192 | 291 | 231 | 1161 | 1392 | 80 | 4 | 314 | 5 | 1683 | 279 | 2287 | 1784 |
| 1000 nM | 184 | 307 | 173 | 1075 | 1248 | 69 | 2 | 267 | 4 | 1477 | 231 | 2087 | 1576 |
| 3160 nM | 214 | 305 | 123 | 862 | 985 | 55 | 2 | 188 | 2 | 1208 | 243 | 1841 | 1312 |
| 10,000 nM | 180 | 300 | 78 | 499 | 577 | 93 | 2 | 186 | 1 | 862 | 282 | 1472 | 976 |
|  | **PATIENT AML218 AVERAGE COUNTS** | | | | | | | | | | | | |
| DMSO (0 nM) | 2637 | 841 | 96 | 1424 | 1520 | 3503 | 54 | 4974 | 38 | 6046 | 3426 | 12265 | 8690 |
| 32 nM | 2748 | 836 | 141 | 1854 | 1995 | 1033 | 28 | 1163 | 25 | 3967 | 1469 | 8267 | 4569 |
| 100 nM | 3001 | 886 | 321 | 2651 | 2972 | 837 | 12 | 1103 | 27 | 4298 | 1142 | 9023 | 5034 |
| 317 nM | 3119 | 880 | 287 | 3107 | 3394 | 773 | 7 | 1170 | 21 | 4452 | 1021 | 9365 | 5275 |
| 1000 nM | 3073 | 893 | 305 | 2950 | 3255 | 780 | 11 | 1055 | 31 | 4277 | 1016 | 9184 | 5146 |
| 3160 nM | 3062 | 884 | 330 | 2768 | 3098 | 750 | 8 | 1049 | 20 | 4116 | 1026 | 8971 | 4963 |
| 10,000 nM | 2856 | 889 | 178 | 2424 | 2602 | 789 | 12 | 931 | 26 | 3855 | 1316 | 8557 | 4785 |
|  | **PATIENT AML224 AVERAGE COUNTS** | | | | | | | | | | | | |
| DMSO (0 nM) | 19 | 310 | 6 | 5 | 11 | 199 | 3 | 413 | 109 | 25279 | 25715 | 26529 | 26117 |
| 32 nM | 28 | 233 | 14 | 1 | 15 | 38 | 0 | 258 | 20 | 12457 | 12960 | 13381 | 13085 |
| 100 nM | 7 | 274 | 0 | 1 | 1 | 54 | 0 | 159 | 4 | 8345 | 8837 | 9270 | 8962 |
| 317 nM | 11 | 259 | 5 | 1 | 6 | 48 | 0 | 112 | 3 | 7531 | 7982 | 8395 | 8095 |
| 1000 nM | 8 | 255 | 0 | 0 | 0 | 25 | 0 | 100 | 2 | 7389 | 7844 | 8217 | 7926 |
| 3160 nM | 10 | 255 | 0 | 1 | 1 | 39 | 0 | 129 | 2 | 7252 | 7735 | 8123 | 7829 |
| 10,000 nM | 14 | 237 | 3 | 1 | 4 | 13 | 0 | 122 | 4 | 8208 | 8844 | 9186 | 8907 |
|  | **PATIENT AML237 AVERAGE COUNTS** | | | | | | | | | | | | |
| DMSO (0 nM) | 240 | 418 | 27 | 4336 | 4363 | 951 | 7 | 2088 | 27 | 12879 | 8330 | 14738 | 13834 |
| 32 nM | 223 | 413 | 98 | 4710 | 4808 | 63 | 9 | 2570 | 20 | 6385 | 1793 | 7444 | 6759 |
| 100 nM | 188 | 410 | 172 | 5763 | 5935 | 34 | 16 | 3412 | 20 | 6818 | 1099 | 7756 | 7126 |
| 317 nM | 190 | 421 | 207 | 5396 | 5603 | 24 | 3 | 2116 | 16 | 6303 | 903 | 7232 | 6586 |
| 1000 nM | 181 | 350 | 95 | 4958 | 5053 | 16 | 4 | 2571 | 15 | 5709 | 861 | 6564 | 5996 |
| 3160 nM | 186 | 386 | 101 | 4478 | 4579 | 20 | 2 | 1522 | 15 | 5294 | 932 | 6216 | 5600 |
| 10,000 nM | 173 | 406 | 54 | 4211 | 4265 | 19 | 1 | 1620 | 21 | 5020 | 1095 | 6065 | 5433 |
|  | **PATIENT AML238 AVERAGE COUNTS** | | | | | | | | | | | | |
| DMSO (0 nM) | 819 | 275 | 0 | 256 | 256 | 22 | 21 | 987 | 23 | 2049 | 2262 | 3766 | 2564 |
| 32 nM | 819 | 296 | 0 | 269 | 269 | 31 | 17 | 837 | 18 | 1745 | 1837 | 3369 | 2167 |
| 100 nM | 819 | 275 | 0 | 304 | 304 | 27 | 22 | 698 | 22 | 1620 | 1720 | 3246 | 2078 |
| 317 nM | 921 | 269 | 0 | 304 | 304 | 27 | 19 | 730 | 23 | 1693 | 1768 | 3407 | 2126 |
| 1000 nM | 891 | 263 | 0 | 277 | 277 | 24 | 23 | 672 | 25 | 1601 | 1731 | 3293 | 2059 |
| 3160 nM | 887 | 286 | 0 | 229 | 229 | 19 | 11 | 574 | 15 | 1469 | 1632 | 3139 | 1899 |
| 10,000 nM | 896 | 279 | 0 | 173 | 173 | 20 | 15 | 544 | 17 | 1438 | 1646 | 3109 | 1858 |

**Supplementary Table 2. Summary of responses to emvododstat**

|  | **Patient Sample** | | | | |
| --- | --- | --- | --- | --- | --- |
| **Parameter** | **42** | **218** | **224** | **237** | **238** |
| CC_50_ Total blasts^a^ | 24 nM | > 10 µM | 32 nM | 32 nM | > 10 µM |
| CC_50_ Immature blasts^a^ | 20 | 28 | 32 | 20 | > 10 µM |
| % Increase in total CD14+ cells at 100 nM emvododstat | 44 | 96 | No increase | 36 | 19 |
| % Blasts pretreatment | 93 | 49 | 95 | 87 | 54 |
| % Blasts posttreatment at 100 nM | 74 | 48 | 90 | 88 | 50 |
| Response to emvododstat | Cytotoxicity & differentiation | Differentiation | Cytotoxicity | Cytotoxicity & differentiation | Resistance |

^a^ The CC_50_ was calculated by assuming the percent inhibition in blast number increased linearly from 0% to 50%.
